# Five-Year Follow-Up of Concomitant Accelerated Hypofractionated Radiation in Advanced Squamous Cell Carcinoma of the Buccal Mucosa: A Retrospective Cohort Study

**DOI:** 10.1155/2015/963574

**Published:** 2015-05-14

**Authors:** Hassan Iqbal, Arif Jamshed, Abu Bakar Hafeez Bhatti, Raza Hussain, Sarah Jamshed, Muhammad Irfan, Natasha Hameed, Adeel Illyas

**Affiliations:** ^1^Department of Surgical Oncology, Shaukat Khanum Memorial Cancer Hospital and Research Centre, Lahore, Pakistan; ^2^Department of Radiation Oncology, Shaukat Khanum Memorial Cancer Hospital and Research Centre, Lahore, Pakistan; ^3^Aga khan University, Karachi, Pakistan; ^4^Lahore Medical and Dental College, Lahore, Pakistan

## Abstract

In resource limited settings, induction chemotherapy with Gemcitabine and Cisplatinum and concurrent hypofractionated chemoradiation for locally advanced carcinoma of buccal mucosa (BMSCC) are a cost effective option but remain under reported. The objective of this study was to report long term survival outcome after concurrent hypofractionated radiotherapy in locally advanced BMSCC. Between February 2005 and 2009, 63 patients received treatment. Induction chemotherapy (IC) regimen consisted of two drugs: Gemcitabine and Cisplatin. All patients received 55 Gy of radiation in 20 fractions with concurrent single agent Cisplatin (75 mg/m^2^). Five-year overall survival (OS), disease-free survival (DFS), and progression-free survival (PFS) were determined. Based on AJCC staging, 7 (11%) patients were stage III, 31 (49%) stage IV a, and 25 (40%) stage IVb at presentation. After IC, 8 (18%) patients had complete radiological response, 33 (73%) had partial response, and 4 (9%) had stable disease. After concurrent hypofractionated chemoradiation, thirty-nine (62%) patients were complete responders and 24 (38%) had stable disease. With a minimum follow-up of 60 months, 5-year OS, DFS, and PFS were 30%, 49%, and 30%, respectively. In locally advanced buccal mucosa squamous cell carcinoma, concurrent hypofractionated chemoradiation results in acceptable survival and regimen related toxicity.

## 1. Introduction

Oral cancer is uncommon in the West but far more prevalent in Asian countries like India and Taiwan where it is strongly associated with betel quid chewing [1]. Among all subsites, an alarming 30–40% of intraoral malignancies arise from buccal mucosa. Surgery followed by radiation remains the treatment of choice [2]. Tortuous anatomy of buccal space combined with the aggressive nature of this malignancy resists surgical attempts and results in poor prognosis in advanced cases. In treatment of locoregionally advanced head and neck cancer, chemoradiation (CRT) has shown superior results when compared with radiation (RT) alone [3]. Comparable results have been demonstrated with altered fractionation and conventional chemoradiation [4, 5]. Single agents like carboplatin and methotrexate have been added to hypofractionated radiation protocol demonstrating comparable results and acceptable toxicity [6, 7]. In developing countries with limited resources and large patient burden hypofractionation allows an efficient use of resources. We used hypofractionated radiation (55 Gy/2.75 Gy per day, completed in 20 days, administered 5 days a week) instead of the standard where 70 Gy is administered over 35 days, 5 days a week. Hypofractionation allowed a shortened stay and early return back home for patients who had affordability issues. In addition, it required 2 cycles of concurrent Cisplatin versus standard 3 cycles since radiation was completed before the third cycle was due. This potentially lowered chemotherapy related toxicity. The aim of current study was to report survival outcomes at 5 years of follow-up with hypofractionated radiation and concurrent single agent chemotherapy in the treatment of locally advanced BMSCC.

## 2. Methods

We retrospectively reviewed 63 patients who presented between February 2005 and February 2009 with locally advanced, histologically verified buccal mucosa squamous cell carcinoma (BMSCC) and were treated with curative intent at the head and neck clinic in Shaukat Khanum Memorial Cancer Hospital and Research Center. Patients included in this study had inoperable disease. Exclusion criteria included all the patients treated with radiation only, patients undergoing surgery as part of their treatment protocol, or patients presenting with metastatic disease at the time of presentation.

Age ranged from 24 to 77 years (median 52 years). Pretreatment evaluation included clinical examination, MRI face and neck, chest X-rays, Orthopantomogram (OPG), complete blood profile, serum electrolytes, and liver and renal function tests. Tumors were staged in accordance with the guidelines set by the American Joint Committee on Cancer staging system (AJCC) 6th edition. All patients underwent pretreatment dental examination and those with signs of widespread and/or advance periodontitis had tooth extractions prior to the commencement of chemoradiotherapy (CRT). Patients with trismus and/or those undergoing chemotherapy were provided nutritional support via percutaneous endoscopic gastrostomy (PEG).

### 2.1. Induction Chemotherapy

**Figure 1:**
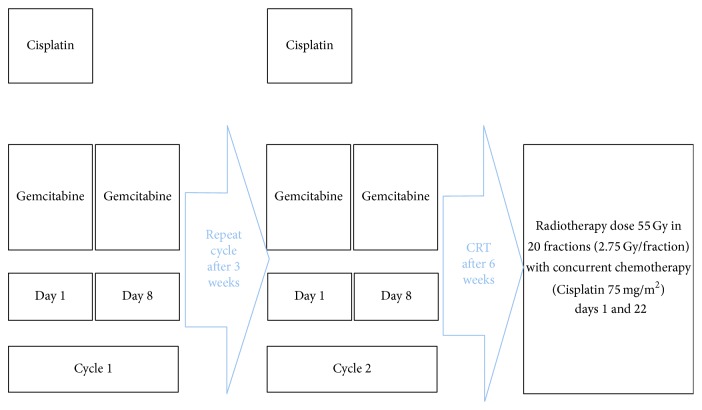
Treatment protocol.

Induction chemotherapy was administered on outpatient basis. The indications for induction chemotherapy included bulky disease, inoperable disease (tumors in which gross clear margin was difficult to achieve), and tumors extending into submasseteric space. Regimen consisted of a combination of 2 drugs, intravenous Gemcitabine 1000 mg/m^2^ on day 1 and day 8 and Cisplatin 75 mg/m^2^ on day 1 of each cycle, respectively (Figure 1). A three-week interval was observed between the 2 cycles. Two weeks after completion of second cycle, a response assessment was clinically devised. A total of 45 patients (71%) were administered IC before chemoradiation. For analytical purposes, response assessment was graded in accordance with the NCI response criteria.

### 2.2. Radiotherapy

All patients underwent simulation and received a total dose of 55 Gy in 20 fractions at 2.75 Gy per fraction for five days a week. Radiotherapy was administered on either cobalt-60 or 6-MV linear accelerator with opposing anterior-posterior portals. The spinal cord was excluded after 30.25 Gy with shrinking field technique. Primary site was treated with a 2 cm clearance margin along with ipsilateral neck up to the lower border of the clavicle. Cone-down was done to exclude the spinal cord after 30.25 Gy. After cone-down, the gross disease was treated all the way. Single agent Cisplatin 75 mg/m^2^ was concurrently administered on days 1 and 22 in all patients. The severity of toxicity related to concurrent chemoradiation was graded according to common toxicity criteria (CTC).

### 2.3. Statistical Analysis

All statistical analysis was performed using SPSS (Statistical Package for the Social Sciences) version 19. A *P* value < 0.05 was considered statistically significant. Kaplan Meier curves were used to determine overall survival (OS), progression-free survival (PFS), and disease-free survival (DFS). Time for OS was calculated by subtracting date of last followup/death from date of biopsy and for DFS by subtracting date of relapse from the date of biopsy. For patients with residual disease clinically/radiologically, PFS was calculated from date of biopsy to date of progression. The hospital ethics committee granted exemption from formal review of this study.

## 3. Results

### 3.1. Patient Demographics

Table 1 demonstrates demographic traits of study population. Male to female ratio was 2.3 : 1. Betel nut chewing was positive in 29 (46%) patients. Nodal involvement was more frequent with T4 tumors (43% versus 3%).

### 3.2. Toxicity

Summary of acute toxicity related to chemoradiation is shown in Table 2. There were no treatment related deaths and no patient developed grade 4 toxicity related to either chemotherapy or radiation. A total of four patients required hospital admission related to toxic effects of chemotherapy. Severe renal impairment (grade 3) was seen in 1 (2%) patient. Two patients were admitted due to febrile neutropenia and 1 patient was admitted due to diarrhea and vomiting. There were no toxicity related deaths.

### 3.3. Response to Treatment

Table 3 demonstrates response to IC and hypofractionated concurrent chemoradiation. A total of 45 patients received IC. After completion of IC, 8 (18%) patients had complete response, 33 (73%) had partial response, and 4 (9%) showed stable disease. After completion of concurrent hypofractionated chemoradiation, 39 (62%) patients were complete responders and 24 (38%) had persistent/progressive disease. Majority of patients with persistent/progressive disease at the completion of the treatment protocol had pretreatment nodal involvement.

### 3.4. Failures

Table 4 demonstrates patterns of relapse. A total of 19 (48.7%) patients relapsed after a complete clinical response in 39 patients. Salvage surgery was performed in 6 patients of whom 4 had complete pathological response on histology while 2 had persistent disease.

### 3.5. Survival

**Figure 2:**
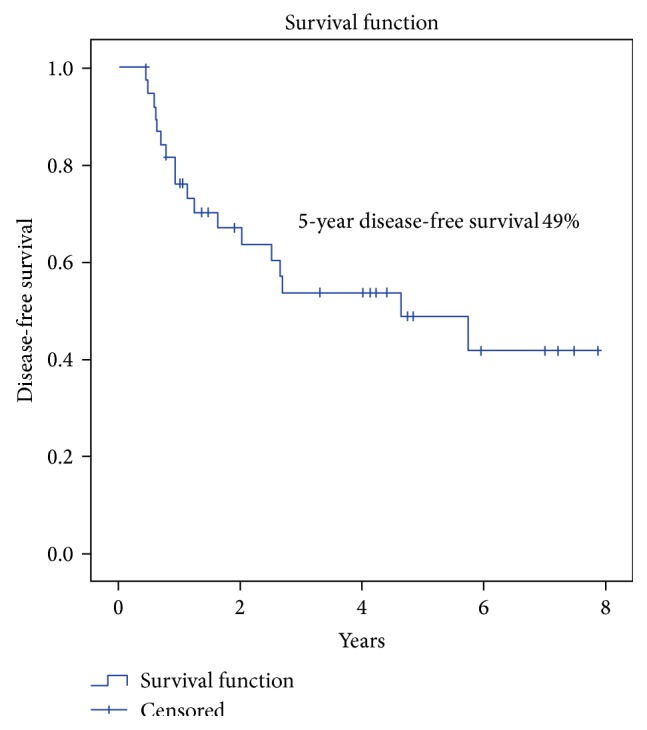
Disease-free survival (DFS) in patients with locally advanced BMSCC.

**Figure 3:**
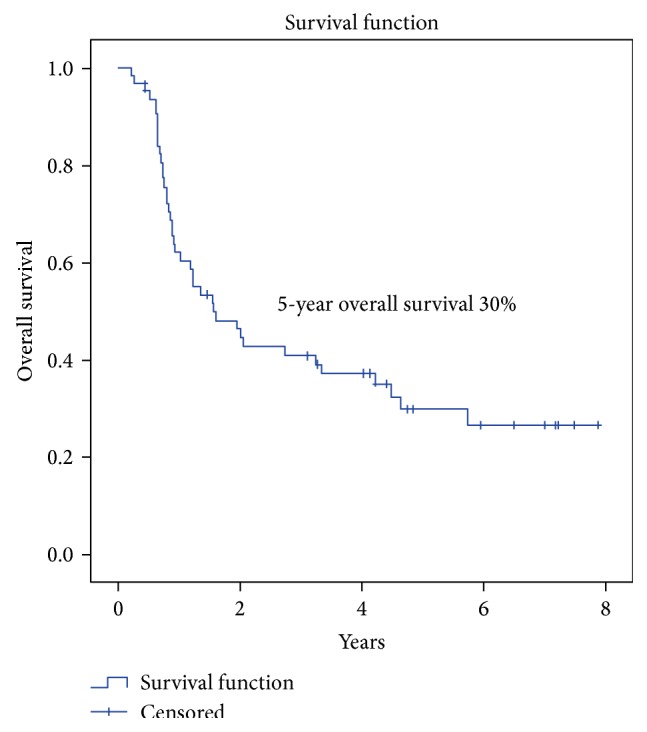
Overall survival (OS) in patients with locally advanced BMSCC.

**Figure 4:**
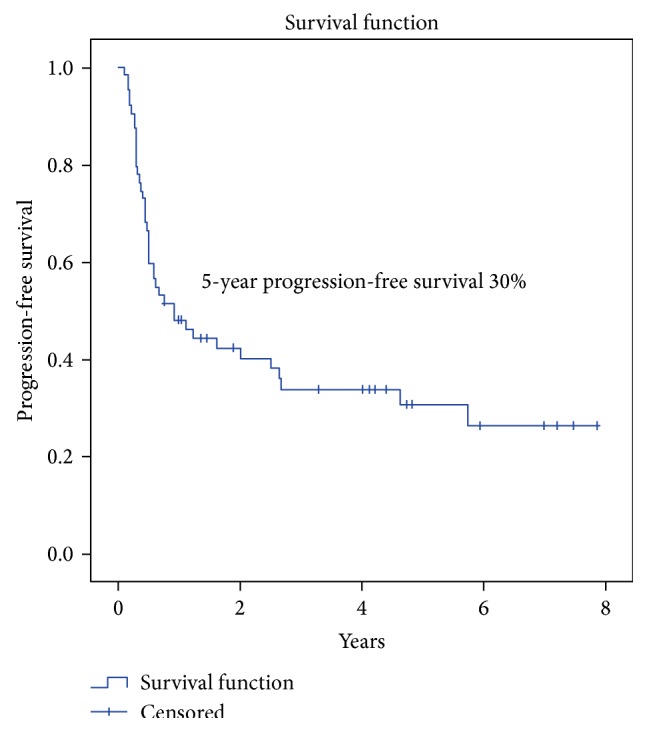
Progression-free survival (PFS) in patients with locally advanced BMSCC.

The OS and DFS were 30% and 49% at 5 years (Figures 2 and 3). Grade and nodal status were the only statistically significant prognostic factors with respect to OS (Table 5). Progression-free survival of the whole group at 5 years was 30% (Figure 4). The 5-year local control, regional control, and locoregional control were 58%, 84%, and 90%, respectively.

## 4. Discussion

In comparison with West where smoking is more common, betel nut chewing was the most common risk factor in the current study. Outcomes were reported based on actual 5-year followup of patients treated with concurrent hypofractionated radiation in a country with resource limited settings. A high percentage of patients presented with nodal involvement at the time of presentation. Gemcitabine based regimen with lower dose settings resulted in acceptable toxicity and good compliance and in combination with hypofractionation resulted in comparable overall and disease-free survival.

Although surgery followed by radiation remains the mainstay treatment in the management of BMSCC, the advent of chemoradiation has diversified options [3–5]. Adequate resection with negative surgical margins and later reconstruction is clearly a challenge with locally advanced BMSCC. The decision to operate or not is dependent on both patient and resource-related factors. Patient-related factors such as unwillingness to consent to surgery, lack of affordability, and medical morbidity may limit surgery from being the treatment of choice. Resource-related factors that dictate operability nest in the availability of multidisciplinary teams for surgery, accessible resources for reconstruction, and the presence of rehabilitation centers. In the current study, tumors were generally bulky and infiltrative with evidence of gross tumor invasion into skin and submasseteric space. Thus, radical surgery with clear margins was difficult to achieve.

Various studies have reported outcomes of single modality treatment for BMSCC. Nair and colleagues in their study on 234 patients of BMSCC treated with radiotherapy alone showed DFS of 41% and 15% for stages III and IV, respectively, and concluded that this treatment protocol is dismal [8]. With respect to surgery alone, Bloom and Spiro published their 13-year experience with 121 BMSCC patients. The 5-year determinate cure rate for stages III and IV disease was 27% and 18% [9]. CRT has become the standard of care for locoregionally advanced head and neck cancer with an absolute survival benefit of 8% at 5 years over radiation alone [10]. The 5-year OS and DFS in our study were 30% and 49%. Such a survival rate is not only equivocal when compared with established treatment modalities but is comparatively better considering the fact that 40% of our patients had stage IVb disease.

The role of induction chemotherapy remains controversial and is continuously evolving for the last 3 decades. Although many phase III trials have shown that use of induction chemotherapy has no overall survival benefit in HNSCC, newer studies have shown a decrease in the incidence of distant metastasis and have opened new avenues for organ preservation [11–13]. We used induction primarily for two reasons. One is the advanced stage at which tumors generally presented to us with skin and muscle involvement. Induction chemotherapy not only melts the tumor but also reduces the pain and improves mouth opening and oral intake. Reducing the tumor size also reduces the necrotic tissue in oral cavity and thus improves the oral hygiene and overall performance status of patients for concurrent radiation or chemoradiation. Other advantage of induction was selection of patients that were responsive to chemotherapy.

Our study showed that none of the patients who received induction chemotherapy developed distant metastasis. The debate for optimization of drugs for induction chemotherapy is far from over. Cisplatin and fluorouracil are the most extensively studied regimens. Despite high response rates, these regimens carry severe side effects [14]. The TAX 324 and TAX 323 trials have compared a two-drug regimen (Cisplatin and fluorouracil) with three-drug regimen (Cisplatin, fluorouracil, and docetaxel) and have shown later to be superior in terms of overall survival [15, 16]. In a developing country like Pakistan, huge tumor burden, limited treatment facilities, and affordability make it necessary to develop cheaper regimens with shorter duration of administration without compromising outcomes. A few phase II trials evaluated the chemotherapeutic activity of Gemcitabine in recurrent metastatic carcinoma of head and neck [17, 18]. Hitt and colleagues included 24 patients with recurrent and metastatic disease and 22.7% overall response rate was observed. They concluded that Cisplatin plus Gemcitabine combination has an acceptable toxicity profile in patients with recurrent and metastatic head and neck cancer [18]. Jamshed and colleagues in their study on treatment of locoregionally advanced nasopharyngeal carcinoma have used Gemcitabine and Cisplatin as induction chemotherapy. They have shown that 15% of patients had complete response after induction chemotherapy with good patient compliance and acceptable toxicity [19]. In the present study Gemcitabine was given in combination with Cisplatin as induction chemotherapeutic drug, which showed complete response in 18% patients. A recent study published has shown that Gemcitabine can induce host antitumor immune response that could facilitate antitumor effects in oral cancer [20]. In the current study, no chemotherapy related deaths were observed and only 4 patients required hospital admission. A lower than standard dose of induction Gemcitabine and concurrent Cisplatin probably resulted in lower toxicity in current study.

In the conventional protocol for head and neck cancer, 70 Gy of radiation is delivered in 7 weeks at 2 Gy per day single fraction. Substantial data have unraveled that tumors of oral cavity undergo accelerated repopulation in the 4th week of conventional radiotherapy and thus require higher doses to overcome this undesired effect [21]. Hypofractionated concurrent radiotherapy in combination with chemotherapy has not been proven to provoke any substantial posttreatment tissue repopulation. Hypofractionated radiotherapy treatment is completed before accelerated repopulation becomes significant. Benefits of addition of chemotherapy to hypofractionated regimen are yet to be proven and dose optimized. Abrahim-al-Mamgani and collegaues treated 158 patients of advanced HNSCC with hypofractionated radiotherapy. They concluded that hypofractionated radiotherapy can be used for local and symptomatic control in palliative setting for advanced head and neck tumors. Sanghera and colleagues radically treated head and neck tumors with hypofractionated radiation and concurrent chemotherapy. Although only 7.4% of the patients had oral cavity tumors, OS for the whole group at 5 years was close to 50% [22].

On the other end of the spectrum, hyperfractionated radiotherapy has gained considerable popularity but the resource intense schedule is not feasible in resource limited settings [23]. Large fraction size in the hypofractionated radiotherapy regimen has been criticized for the development of late toxicity. Fowler compared conventional RT and hypofractionated RT and reported lower rate of late toxicity with hypofractionated regimen albeit comparable local control [24, 25]. Chan and colleagues in their study showed a 2-year OS of 50% for tumors of oral cavity treated with hypofractionated RT and concurrent carboplatin [26]. In the current study the OS and DFS of the whole group at 2 years were 42% and 64%, respectively. Although late toxicity related to radiotherapy was not formally documented in our study, patients tolerated the hypofractionated RT well with concurrent chemotherapy. A PEG tube was inserted in 35/63 patients prior to the start of the treatment. Duration of radiation ranged between 26 and 42 (mean 28) days. The reason for radiation completion in <28 days was that patients came from all over the country and due to logistics problems treatment was completed over the weekends. In only 10 patients treatment completion required more than 30 days secondary to mechanical breakdown of machines. Also, we did not observe any hospital admissions related to radiation induced toxicity. Limitations of our study included retrospective design and lack of the documentation of late toxicity of radiation.

Gemcitabine and Cisplatin were well tolerated and showed acceptable response with hypofractionated chemoradiation. Our disease-free survival and overall survival were comparable to outcomes reported with standard chemoradiation protocols. It is important to remember that the minimum followup in the current study was 5 years. The routine of hypofractionation combined with low dose Cisplatin allowed early completion of treatment and acceptable toxicity due to lower dose of concurrent Cisplatin. This might represent an effective treatment option in resource limited settings. However, validation in a randomized trial is warranted to confirm its applicability.

## Figures and Tables

**Table 1:** Patient characteristics.

	Number *N* = 63	Percent (%)
Age (years)		
<40	07	11
>40	56	89
Sex		
Male	44	70
Female	19	30
ECOG (performance status)		
0	11	18
1	52	82
Risk factors		
Smoking	27/63	43
Pan (betel nut)	29/63	46
Naswar (tobacco chew)	17/63	27
Alcohol	0	0
No risk factors	9/63	14
Grade		
Well (G1)	44	70
Moderate (G2)	15	24
Poor (G3)	04	6
Stage		
III	05	8
IVa	33	52
IVb	25	40
T3		
N0	05	7
N+	02	3
T4		
N0	29	46
N+	27	43
Bone invasion	39	62
Retromolar trigone invasion	07	11
Percutaneous gastrostomy		
Yes	35	56
No	28	44
Submasseteric space	25	40
Extractions of teeth	40	64

**Table 2:** Acute toxicity with induction chemotherapy and chemoradiation.

	G0		G1		G2		G3		G4	
	Number	Percent	Number	Percent	Number	Percent	Number	Percent	Number	Percent
Anemia	58	92	4	6	1 (2)	2	—		—	—
Neutropenia	34	54	5	8	10	16	4	6	—	—
Thrombocytopenia	54	86	7	11	1	2	1	2	—	—
Vomiting	57	90	3	5	2	3	1	2	—	—
Diarrhea	59	94	1	2	2	3	1	2	—	—
Creatinine	54	86	8	13	—	—	1	2	—	—
Hyperbilirubinemia	63	100	—	—	—	—	—	—	—	—
Fever	61	97	2	3	—	—	—	—	—	—
Mucositis	—	—	23	36	32	51	8	13	—	—

**Table 3:** Response to treatment.

Response after Induction chemotherapy	Number *N* = 63	Percent (%)	Number *N* = 63	Percent (%)	Number *N* = 63	Percent (%)
	CR		PR		SD/PD	
Local						
T3	—	—	4	9	—	—
T4	8	18	29	64	4	9
Regional						
N0	4	9	15	34	2	4
N+	4	9	18	40	2	4
Stage						
III	—	—	4	9		
IV	8	18	29	64	4	9
Response 6 weeks after completion of the treatment						
T3	5	10	—	—	2	3
T4	34	54	—	—	22	35
Regional						
N0	26	42	—	—	8	13
N+	13	20	—	—	16	25
Stage						
III	3	5	—	—	2	3
IV	36	57	—	—	22	35

CR: complete response; PR: more than 50% reduction; SD: less than 50% reduction; PD: persistent/progressive disease.

**Table 4:** Patterns of failure in patients with complete clinical response.

Recurrence site	Number	Percent
	*N* = 19	(%)
Local	13	21
Regional		
Ipsilateral	1	6
Contralateral	3	
Locoregional		
Ipsilateral	2	3

**Table 5:** Influence of prognostic factors on overall survival.

Prognostic indicator	5-year overall survival (%)	*P* value
Age		
<40	58	0.1
>40	22	
Gender		
Male	30	0.3
Female	22	
Stage		
III	28	0.5
IV	29	
Grade		
Well	38	0.03
Moderate	10	
Poor	22	
Bone invasion		
Yes	31	0.6
No	28	
Submasseteric space involvement		
Yes	38	0.9
No	25	
Retromolar trigone involvement		
Yes	12	0.5
No	32	
Nodal status		
N0	38	0.01
N+	16	
Induction chemotherapy		
Yes	22	0.2
No	40	
